# Genome-wide identification of *NHX* (Na^+^/H^+^ antiporter) gene family in *Cucurbita L.* and functional analysis of *CmoNHX1* under salt stress

**DOI:** 10.3389/fpls.2023.1136810

**Published:** 2023-03-14

**Authors:** Changwei Shen, Jingping Yuan, Xin Li, Ruixiang Chen, Daohan Li, Fei Wang, Xing Liu, Xinzheng Li

**Affiliations:** ^1^School of Resources and Environmental Sciences, Henan Institute of Science and Technology, Xinxiang, China; ^2^School of Horticulture and Landscape Architecture, Henan Institute of Science and Technology, Xinxiang, China; ^3^Henan Engineering Research Center of the Development and Utilization of Characteristic Horticultural Plants, Xinxiang, China

**Keywords:** *Cucurbita* L., Na^+^/H^+^ antiporter, evolutionary relationship, *NHX1*, expression pattern

## Abstract

Soil salinization, which is the accumulation of salt in soil, can have a negative impact on crop growth and development by creating an osmotic stress that can reduce water uptake and cause ion toxicity. The NHX gene family plays an important role in plant response to salt stress by encoding for Na^+^/H^+^ antiporters that help regulate the transport of sodium ions across cellular membranes. In this study, we identified 26 *NHX* genes in three cultivars of *Cucurbita* L., including 9 *Cucurbita moschata NHXs (CmoNHX1-CmoNHX9)*, 9 *Cucurbita maxima NHXs* (*CmaNHX1-CmaNHX9*) and 8 *Cucurbita pepo NHXs* (*CpNHX1-CpNHX8*). The evolutionary tree splits the 21 *NHX* genes into three subfamilies: the endosome (Endo) subfamily, the plasma membrane (PM) subfamily, and the vacuole (Vac) subfamily. All the *NHX* genes were irregularly distributed throughout the 21 chromosomes. 26 *NHXs* were examined for conserved motifs and intron-exon organization. These findings suggested that the genes in the same subfamily may have similar functions while genes in other subfamilies may have functional diversity. The circular phylogenetic tree and collinearity analysis of multi-species revealed that *Cucurbita* L. had a substantially greater homology relationship than *Populus trichocarpa* and *Arabidopsis thaliana* in terms of *NHX* gene homology. We initially examined the *cis*-acting elements of the 26 *NHXs* in order to investigate how they responded to salt stress. We discovered that the *CmoNHX1*, *CmaNHX1*, *CpNHX1*, *CmoNHX5*, *CmaNHX5*, and *CpNHX5* all had numerous ABRE and G-box *cis*-acting elements that were important to salt stress. Previous transcriptome data showed that in the mesophyll and veins of leaves, many *CmoNHXs* and *CmaNHXs*, such as *CmoNHX1*, responded significantly to salt stress. In addition, we heterologously expressed in *A. thaliana* plants in order to further confirm the response of *CmoNHX1* to salt stress. The findings demonstrated that during salt stress, *A. thaliana* that had *CmoNHX1* heterologously expression was found to have decreased salt tolerance. This study offers important details that will aid in further elucidating the molecular mechanism of *NHX* under salt stress.

## Introduction

Soil salinization is a serious problem that can have negative impacts on plant growth and development, and it is becoming an increasingly severe global environmental issue. The area of salinized soil is expanding due to a combination of natural environmental changes, such as climate change and changes in water availability, as well as human activities like improper irrigation practices, deforestation, and urbanization. One of the key problems impeding the sustainable growth of agriculture is soil salinization, which has emerged as a severe global environmental issue (Zhu, 2016). The negative effects of salt, which gravely endanger plant growth and development, extend to more than 800 million hectares of land worldwide (Munns and Tester, 2008). The area of salinized soil exhibits a trend of progressive increase under the simultaneous effects of natural environment changes and human activity (Munns and Tester, 2008). China is one of the countries that is particularly affected by soil salinization. According to Qi et al. (2012), China has approximately 36 million hectares of salinized agricultural land, which accounts for around 5% of the country’s total agricultural land. This can have significant implications for food security and agricultural sustainability in China, as crops grown in these areas may experience reduced yields or even failure due to salt stress.

In the process of plant growth, Na^+^ is a non-essential element. The roots of plants can absorb a lot of the highly mobile Na^+^ in salinized soil, which can subsequently be transported to the cytoplasm of various tissues and organs to cause salt damage (Rajendran et al., 2010; Roy et al., 2014). Na^+^ homeostasis in plants is largely maintained by genes from the Na^+^/H^+^ antiporter family. Eight *NHX* genes were discovered in *Arabidopsis thaliana*, which were separated into the vacuole (Vac) subfamily, the plasma membrane (PM) subfamily, and the endosome nucleolus (Endo) subfamily (Ohnishi et al., 2005; Bassil et al., 2011). All *AtNHX* genes contain 10-12 transmembrane structures and participate in various biological processes under salt stress (Ohnishi et al., 2005; Bassil et al., 2011). Seven *PtNHX* genes were identified from *Populus tomentosa* Carr genome (Tian et al., 2017), and they respond to single or multiple stresses such as drought, heat, cold, salt and ABA. *PtNHX7* was involved in salt stress response through calcineurin B-like proteins - calcineurin B-like interacting protein kinase (CBL-CIPK) pathway (Tian et al., 2017). *NHX* gene—*PeSOS1* was found to enhance salt tolerance in *Poplus trichocarpa* (Meng and Wu, 2018). *BvNHX5* may interact with CBL and CIPK to enhance salt tolerance in *Beta vulgaris* (Wu et al., 2019). A total of 25 *NHX* genes were identified in *Gossypium herbaceum*, and *GbNHX2* gene was highly expressed under high salt stress. (Akram et al., 2020). These results suggest that *NHX* genes may play an important role under salt stress in crops.

The *Cucurbita* genus primarily consists of three crop species: *Cucurbita moschata*, *Cucurbita maxima*, and *Cucurbita pepo*, which are widely planted worldwide due to their excellent disease resistance, established roots, and adaptability (Montero-Pau et al., 2018). The main reason why *Cucurbita* genus crops, especially *Cucurbita moschata*, are the main rootstocks of melon crops is that they have salt tolerance and can enhance the stress resistance of melon crops. Na^+/^H^+^ antiporter family genes play an important role in maintaining Na^+^ homeostasis in plants. Since the genomes of three domesticated species of the *Cucurbita* genus were published (Sun et al., 2017), the identification of the Na^+^/H^+^ reverse transporter gene family and their responses to salt stress have not been reported. Herein, this study investigated the evolutionary relationships, gene structures, and regulatory elements of *NHX* genes in three cultivars of the *Cucurbita* genus, and examined the transcriptional changes in two of these cultivars, *Cucurbita moschata* and *C. maxima*, in response to salt stress. We also identified a differentially expressed gene, *CmoNHX1*, which was found to respond to salt stress. To confirm the function of this gene, we genetically modified *Arabidopsis thaliana*, a model plant species, and observed its response to salt stress. These findings have significant implications for developing salt-tolerant cultivars of *Cucurbita* plants, as well as enhancing our understanding of the mechanisms underlying salt tolerance in these plants. By identifying the specific genes involved in salt tolerance, researchers may be able to develop more effective breeding strategies and genetic engineering techniques to enhance salt tolerance in *Cucurbita* crops.

## Materials and methods

### Identification of *NHX* family genes in *Cucurbita*

Eight *NHX* protein sequences of *A. thaliana* were obtained from the TAIR database (https://www.arabidopsis.org/) (Philippe et al., 2012). On this basis, the local BLAST program was used to search for NHX proteins in the database of three cultivars of *Cucurbita* genome (*C. moschata*, *C. maxima*, *C. pepo*) (http://cucurbitgenomics.org/). The NHX candidate proteins must meet the requirements: the protein sequence identity of AtNHX protein with other protein more than 70% and e<10^−10^. Na^+^_H^+^_Exchanger domain (PF00999) of NHX candidate proteins from *Cucurbita* were confirmed by PFAM (http://pfam.xfam.org/) (Finn et al., 2016) and SMART (http://smart.emblheidelberg.de/) (Letunic et al., 2012). Theoretical isoelectric point (*pI*) and molecular weight (*MW*) of NHX candidates were predicted by ExPASy software (https://web.expasy.org/compute\upi/) (Bjellqvist et al., 1993). The TMHMM server v. 2. 0 software (http://www.cbs.dtu.dk/services/TMHMM/) (Moller et al., 2001)was used to predict the membrane domain of all proteins.

### Phylogenetic relationship of NHX family proteins in several species

To elucidate the phylogenetic relationships of *NHX* protein families in *Cucurbita moschata*, *C. maxima*, *C. pepo*, *P. trichocarpa* and *A. thaliana*, NHX proteins from *P. trichocarpa* were firstly extracted from previous literature (Tian et al., 2017). Moreover, NHXs protein sequences in *C. moschata* (*Cmo*), *C. maxima* (*Cma*), *C. pepo* (*Cp*), *P. trichocarpa* (*Pt*) and *A. thaliana* (*At*) were blast by ClustalW program (Larkin et al., 2007). Finally, a circular phylogenetic tree was constructed using MEGA 7.0 software (Kumar et al., 2016) and the Maximum Likelihood method (Guindon and Gascuel, 2003) was applied with bootstrap value set to 1000 replicates.

### Conservative domain and gene structure analysis of NHX family members in *Cucurbita*

To further analyze the conserved domains of *NHX* family members in *Cucurbita*, the MEME suite online program (MEME 5.3.3, http://meme-suite.org/tools/meme) (Bailey et al., 2006) was used to analyze and draw the motifs. The operating parameter were set as: the base width was 6-50 aa and the maximum number of motifs was 15.

To clarify the structural characteristics of *NHX* genes in *Cucurbita*, the CDS sequences of 26 *NHX* genes were compared with the corresponding genomic DNA sequences from the corresponding genomic database. Finally, the Gene Structure Display Server (GSDS, http://gsds.cbi.pku.edu.cn/) (Hu et al., 2014)was used to map exon-intron structure of *NHX* genes.

### Chromosome location and collinearity analysis of *NHXs* in *Cucurbita*

To clarify the distribution of *NHX* gene on chromosomes in *Cucurbita*, we first obtained the starting positions of *CmoNHX*, *CmaNHX*, and *CpNHX* genes on chromosomes from the genome databases of three *Cucurbita cultivars*, and finally the analysis were performed using TBtools (https://github.com/CJ-Chen/TBtools) (Chen et al., 2020).

To further analyze the collinearity of *NHX* genes among *C. moschata* (*Cmo*), *C. maxima* (*Cma*) and *C. pepo* (*Cp*), and their collinearity with *A. thaliana NHX* genes, MCScanX software and Circos-0.69 software (Krzywinski et al., 2009) were used to analyze and visualize the collinearity of *NHX* genes.

### Analysis of *Cis*-acting elements of *NHXs* in *Cucurbita*

To identify the *cis*-acting elements of *NHX* genes in *Cucurbita*, the promoter sequences (1500 bp sequence upstream of start codon) of all *CmoNHXs*, *CmaNHXs* and *CpNHXs* were extracted from the genome of *Cucurbita* (http://cucurbitgenomics.org/). The *cis*-acting elements of these *NHX* genes were then predicted using the PlantCARE website (http://bioinformatics.psb.ugent.be/webtools/plantcare/html/) (Lescot et al., 2002). We analyzed the *cis*-acting elements related to growth and development, hormone and abiotic stress, with emphasis on the *cis*-acting elements related to salt stress.

### Response analysis of *CmoNHXs* and *CmaNHXs* to salt stress

To determine the response of *CmoNHXs* and *CmaNHXs* to salt stress, we excavated the transcriptome data (BioProject: PRJNA464060) published in 2018 (Niu et al., 2018) and analyzed the transcription profile of *NHXs* in the leaf mesophyll and leaf vein of the *C. moschata* cultivar, “*Rifu*” and *C. maxima* cultivar, “*Rimu*” under salt stress. Sequencing samples were obtained from 100 mM NaCl treated for 24 hours. 0 mM NaCl was used as a control. The expression value was calculated in terms of reads per kilobase of exon model per million mapped reads (RPKM).

### Subcellular localization analysis of CmoNHX1 protein

We cloned the *CmoNHX1* gene’s nucleotide sequence (**Table S4**) and inserted it into PRI101-GFP to create the recombinant vector PRI101-GFP-*CmoNHX1* in order to study the subcellular localization of the CmoNHX1 protein. The recombinant vector, control vector PRI101-GFP and plasma membrane marker: pm-rbCD3-1008 were transformed into *Agrobacterium tumefaciens* (GV3101) by freeze-thaw method (Jyothishwaran et al., 2007), respectively. According to the method of Wu et al. (2010), *CmoNHX1* was transiently expressed in tobacco. After 40 hours of dark culture, laser confocal microscopy was used to detect cell fluorescence.

### Construction, genetic transformation and response to salt stress of *CmoNHX1*- ectopic expression vector

To construct *CmoNHX1* ectopic expression vector, we constructed *CmoNHX1* fragment into pTCK303 using recombinant vector PRI101-GFP-*CmoNHX1* as template (**Table S4**), and the recombinant vector was termed as pTCK303-*CmoNHX1*. Transformation of constructed recombinant vector into GV3101 by freeze-thaw method (Jyothishwaran et al., 2007). In addition, transgenic *A. thaliana* plants were obtained by floral dip method (Xu et al., 2010). T2 generation transgenic plants were finally obtained through multi-generation selfing and GUS staining.

We used *A. thaliana* plants that were heterologously expressed in *CmoNHX1* from the T2 generation as well as wild type plants to better understand how *CmoNHX1* responded to salt stress. In order to irrigate the plants at the seedling stage, a 100 mM NaCl solution was made, and 50 mL of it was poured twice, once every three days, into each hole. Water was used as the control. Every time a symptom appeared, pictures were taken. Following the prior procedure, RNA extraction, reverse transcription, and qRT-PCR were carried out at this time. (Yuan et al., 2019).

## Results

### Identification of *NHX* gene family in *Cucurbita*

A total of eight AtNHX protein sequences from *Arabidopsis thaliana* as query sequences were used to identify NHX proteins in three cultivars of the *Cucurbita* genus, including *C. moschata*, *C. maxima*, and *C. pepo*. Using a series of screening procedures, a total of 26 NHX proteins were discovered, which were named *CmoNHX1* through *CmoNHX9*, *CmaNHX1* through *CmaNHX9*, and *CpNHX1* through *CpNHX8*. The naming convention was based on the name of the corresponding *AtNHX* genes and their position on the chromosome, from the first to last chromosomes, and from top to bottom. More information about these NHX proteins can be found in **Table S1** of the study.

Sequence analysis of 26 *NHX* genes showed that the length of open reading frame in 9 *CmoNHXs* ranged from 903 bp (*CmoNHX6*) to 3264 bp (*CmoNHX9*), and the corresponding number of amino acids obtained by translation ranged from 300 aa to 1087 aa (**Table 1**). The *pI* and *MW* of *CmoNHXs* were from 5.41 to 9.13 and from 33.328 98 KDa to 122.789 68 KDa, respectively. Nine *CmoNHXs* contained 5 to 12 transmembrane domains (**Table 1**). **Table 1** also displays the length of the open reading frame, the length of the amino acid, the size of the *pI*, and the *MW* in *CmaNHXs* and *CpNHXs*. These findings accurately capture the diversity and conservation of *NHXs’* biological and structural features.

**Table 1:** Properties and locations of the predicted NHX proteins in *Cucurbita* L.

Species	Nm.	Gene Name	Gene Locus	Chr	Start (bp)	End (bp)	CDs length (bp)	AA length (bp)	Isoelectric point (pI)	Molecular weight (MW)	TM	Orthologous gene ID in Arabidopsis thaliana
*Cucurbita moschata* (Cmo)	1	CmoNHX1	CmoCh01G011470.1	Cmo_Chr01	9406608	9416026	1590	529	8.5	58906.15	11	AT5G55470.1
	2	CmoNHX2	CmoCh04G022490.1	Cmo_Chr04	16821621	16850447	2592	863	5.73	96255.97	12	AT2G01980.1
	3	CmoNHX3	CmoCh08G011760.1	Cmo_Chr08	7482903	7488855	1656	551	6.35	61585.45	9	AT3G05030.1
	4	CmoNHX4	CmoCh10G011690.1	Cmo_Chr10	10438999	10445386	1512	503	8.45	55362.19	10	AT3G05030.1
	5	CmoNHX5	CmoCh11G013070.1	Cmo_Chr11	8965531	8970782	1614	537	6.89	59519.69	10	AT3G05030.1
	6	CmoNHX6	CmoCh13G003410.1	Cmo_Chr13	4368154	4375909	903	300	5.41	33328.98	5	AT1G79610.1
	7	CmoNHX7	CmoCh13G003420.1	Cmo_Chr13	4375945	4381926	1065	354	5.76	38680.79	6	AT1G79610.1
	8	CmoNHX8	CmoCh17G011050.1	Cmo_Chr17	9077906	9086008	1881	626	8.7	70099.28	10	AT3G05030.1
	9	CmoNHX9	CmoCh18G006110.1	Cmo_Chr18	7418948	7432545	3264	1087	9.13	122789.7	8	AT1G79610.1
*Cucurbita maxima*(Cma)	1	CmaNHX1	CmaCh01G011010.1	Cma_Chr01	8073976	8083094	1641	546	9	60212	11	AT5G55470.1
	2	CmaNHX2	CmaCh04G021540.1	Cma_Chr04	15081201	15127514	3429	1142	5.92	126694	12	AT2G01980.1
	3	CmaNHX3	CmaCh08G012010.1	Cma_Chr08	7385483	7391030	1641	546	6.4	60496.75	12	AT3G05030.1
	4	CmaNHX4	CmaCh10G010920.1	Cma_Chr10	7256436	7263128	1614	537	7.25	59235.58	10	AT3G05030.1
	5	CmaNHX5	CmaCh11G012510.1	Cma_Chr11	8327881	8333693	1746	581	8.32	64296.31	9	AT3G05030.1
	6	CmaNHX6	CmaCh13G003230.1	Cma_Chr13	3735510	3743050	861	286	5.39	31854.22	5	AT1G79610.1
	7	CmaNHX7	CmaCh13G003240.1	Cma_Chr13	3743086	3753602	789	262	5.52	28644.79	6	AT1G79610.1
	8	CmaNHX8	CmaCh17G011310.1	Cma_Chr17	7885629	7891787	1611	536	8.63	59850.26	10	AT3G05030.1
	9	CmaNHX9	CmaCh18G006240.1	Cma_Chr18	5526159	5532338	1443	480	6.49	53229.39	8	AT1G79610.1
*Cucurbita pepo* (Cp)	1	CpNHX1	Cp4.1LG02g00760.1	Cp4.1LG02	5117043	5124508	1572	523	7.63	58457.51	9	AT5G55470.1
	2	CpNHX2	Cp4.1LG04g08510.1	Cp4.1LG04	2910144	2916587	1620	539	7.23	59389.56	10	AT3G05030.1
	3	CpNHX3	Cp4.1LG10g12560.1	Cp4.1LG10	9526308	9538085	1425	474	6.75	52149.26	9	AT1G79610.1
	4	CpNHX4	Cp4.1LG11g09280.1	Cp4.1LG11	7755738	7762736	1512	503	8.45	55362.19	10	AT3G05030.1
	5	CpNHX5	Cp4.1LG12g05410.1	Cp4.1LG12	8191682	8201059	1839	612	9.1	68026.99	12	AT3G05030.1
	6	CpNHX6	Cp4.1LG17g00770.1	Cp4.1LG17	13054	16580	1152	383	7.16	42698.38	9	AT3G05030.1
	7	CpNHX7	Cp4.1LG17g00950.1	Cp4.1LG17	590904	596069	1611	536	7.32	59601.61	10	AT3G05030.1
	8	CpNHX8	Cp4.1LG20g06710.1	Cp4.1LG20	4583218	4611386	1959	652	5.43	71147.75	11	AT1G79610.1

Note: TM, the number of transmembrane domains.

### Phylogenetic relationships of NHXs in multiple species

**Figure 1:**
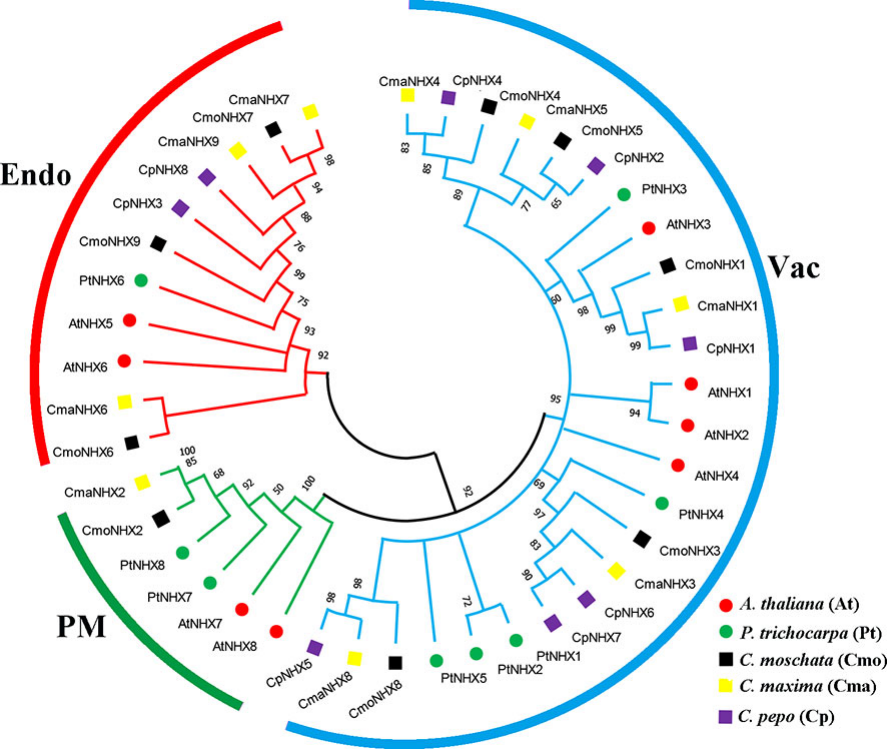
Phylogenetic trees of the *NHX* gene family in a number of species. Three subfamilies (Endo, PM and Vac) were displayed with evolutionary branches of different colors. The phylogenetic tree was constructed with MEGA 7.0 software using the Maximum Likelihood method with 1000 bootstrap replicates. Cmo, *C. moschata*; Cma, *C. maxima*; At, *A. thaliana*; Pt, *P. trichocarpa*.

The study you mentioned constructed a phylogenetic tree of NHX proteins from *Cucurbita moschata*, *C. maxima*, *C. pepo*, *Arabidopsis thaliana*, and *Populus trichocarpa*, to better understand the evolutionary relationships within the plant NHX protein family (**Figure 1**). Based on the amino acid sequence identity of 92%, the NHX proteins were classified into three subfamilies, namely Endo, Vac, and PM. All the NHX proteins were classified into these subfamilies according to the classification of AtNHX proteins. The phylogenetic analysis revealed that the Endo subfamily contained three CmoNHX proteins, three CmaNHX proteins, two CpNHX proteins, two AtNHX proteins, and one PtNHX protein. The PM subfamily included one CmoNHX protein, one CmaNHX protein, and two AtNHX proteins. The Vac subfamily contained the majority of NHX proteins, including five CmoNHX proteins, five CmaNHX proteins, six CpNHX proteins, four AtNHX proteins, and five PtNHX proteins. The study found that the NHX proteins of *Cucurbita*, *Arabidopsis*, and Populus were mostly distributed in the Vac subfamily, followed by the PM subfamily, and finally the Endo subfamily. The PtNHX proteins were mostly found in the Vac subfamily, followed by the Endo subfamily, and lastly the PM subfamily. This information provides insights into the evolutionary history and diversification of NHX proteins in plants. The homology of NHXs among the three domesticated species of *Cucurbita* is significantly higher than that of *A. thaliana* and *P. trichocarpa*, when seen from the perspective of evolutionary lineages (**Figure 1**).

### Analysis of conserved domain of NHX proteins in *Cucurbita*

**Figure 2:**
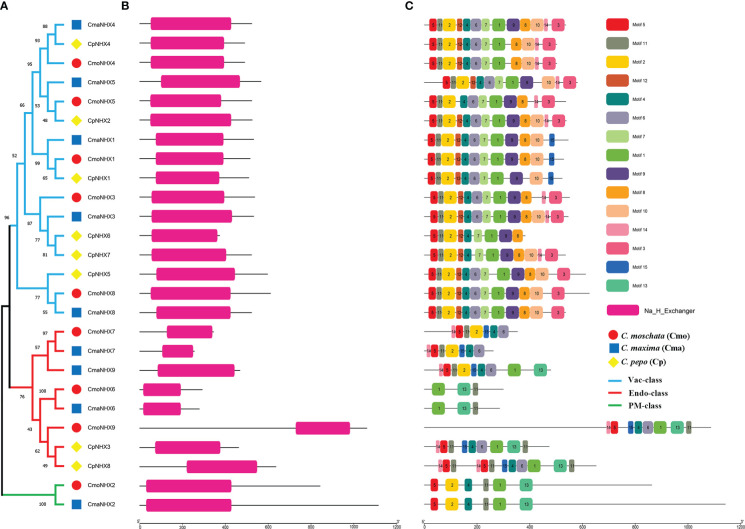
Structural analysis of 26 *Cucurbita* genera NHX proteins. **(A)**, Phylogenetic tree of NHX protein in 26 NHX proteins in *Cucurbita* genus. **(B)**, Conserved domains of 26 NHX proteins in *Cucurbita* genus. **(C)**, Motif analysis of 26 NHX proteins in *Cucurbita* genus.

Twenty-six *Cucurbita* NHX proteins are further grouped into three subfamilies (Endo, PM, and Vac) based on the evolutionary tree (**Figure 2A**). All 26 NHX proteins were found to possess the conserved Na^+^_H^+^_Exchanger domain (PF00999) after conducting a conserved domain analysis (**Figure 2B**). Furthermore, we looked at the motifs of 26 NHX proteins and found a total of 15 motifs (**Figure 2C**; **Figure S2**). Motif 11 was present in all NHX proteins. Furthermore, motif 7 and 12 were exclusive to the Vac subfamily, and motif 13 was present only in the PM and Endo subfamilies (**Figure 2C**). Motifs 5 and 2 are almost always present with motif 11. Overall, PM proteins look very similar to the Vac proteins with some domains missing.

### Intron-exon structure analysis of 26 *Cucurbita NHX* genes

A gene’s biological function is significantly influenced by the intron-exon distribution pattern. All *NHX* genes in the Vac subfamily had 12-16 exons, according to an examination of the intron-exon structure. Exon counts for all *NHX* genes in the Endo subfamily varied greatly (10-24 exons). There were many exons in the *NHX* genes of the PM subfamily (20-23). Similar exon numbers and intron lengths can be seen in genes belonging to the same branch, such as *CmaNHX4* and *CpNHX4*, *CmaNHX1* and *CmoNHX1* (**Figure S1**). However, certain homologous genes clearly display distinct intron-exon structural variations. As an illustration, *CmoNHX2* had 20 exons, but *CmaNHX2* had 23 exons and lengthier introns (**Figure S1**).

### The location distribution of 26 *NHX* genes on chromosomes

**Figure 3:**
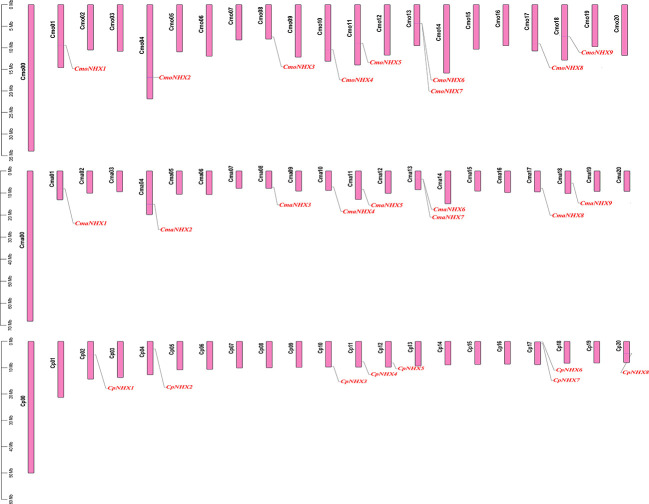
Chromosome distribution map of 26 *NHX* genes in *Cucurbita* genus. Black font represents chromosome name, red font represents gene name.

The distribution of 26 *NHX* genes on the chromosomes revealed that 9 *CmoNHXs* are found on chromosomes Cmo01, Cmo04, Cmo08, Cmo10, Cmo11, Cmo13, Cmo17, and Cmo18 (**Figure 3A**). The distribution pattern of *CmaNHXs* and *CmoNHX* genes is comparable (**Figure 3B**). On the corresponding chromosomes Cmo13 and Cma13, there are two *NHX* genes, respectively (**Figures 3A, B**). The *CpNHX* genes are primarily found on the chromosomes Cp02, Cp04, Cp10, Cp11, Cp12, Cp17, and Cp20 (**Figure 3C**), where two genes are located on chromosome Cp17. Differences between the distribution on Cp vs Cmo/Cma reflect larger differences in chromosome synteny.

### Collinearity analysis of 26 *NHX* genes in *Cucurbita*

**Figure 4:**
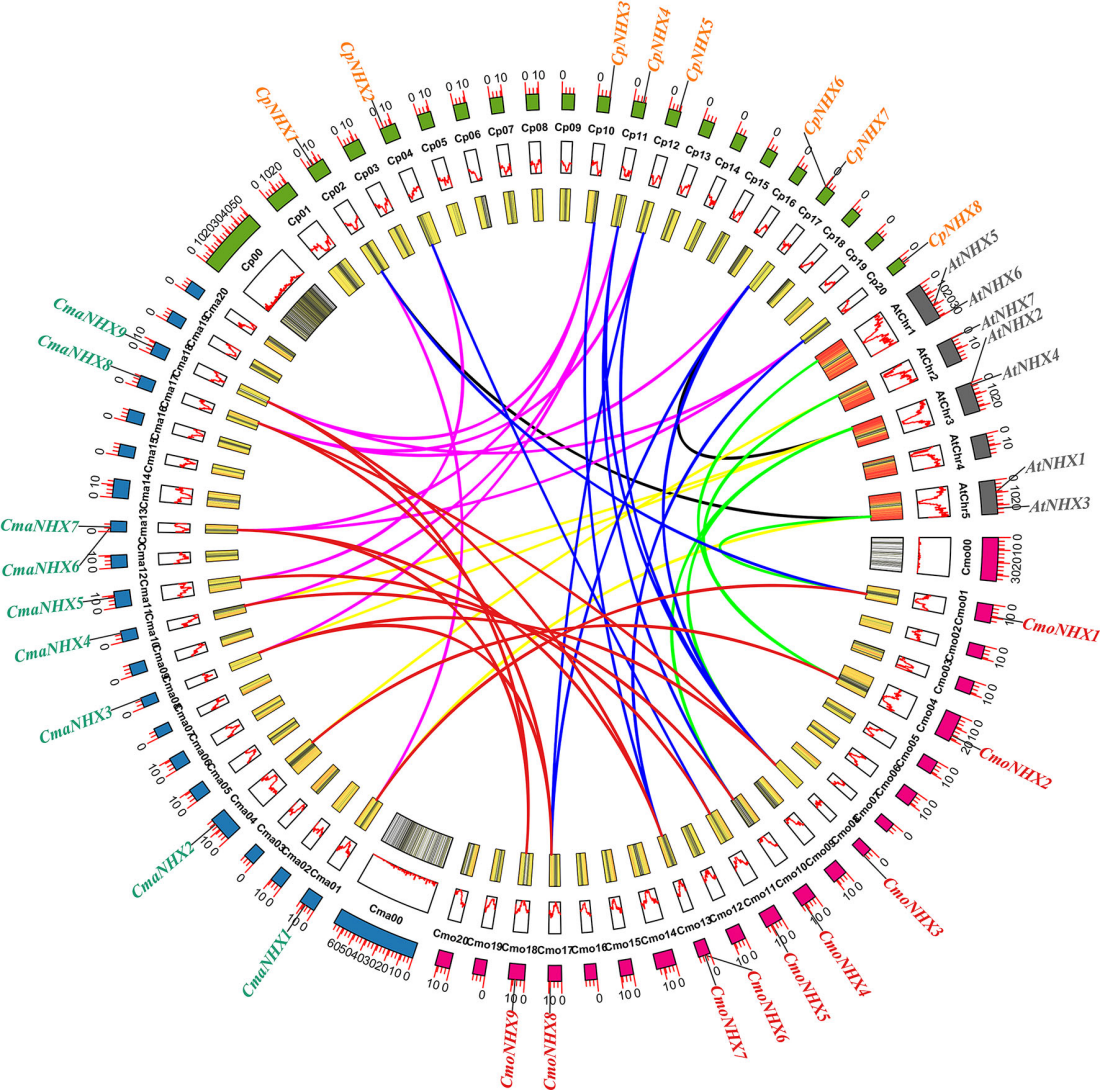
Synteny analysis of the *NHX* genes among *C. moschata*, *C. maxima*, *C. pepo* and *A. thaliana*. Cmo, *C. moschata*; Cma, *C. maxima*; Cp, *C. pepo*; At, *A. thaliana*; The red lines indicated the syntenic *NHX* gene pairs between *C. moschata* and *C. maxima*; The blue lines indicated the syntenic *NHX* gene pairs between *C. moschata* and *C. pepo*; The pink lines indicated the syntenic *NHX* gene pairs between *C. maxima* and *C. pepo*; The green lines indicated the syntenic *NHX* gene pairs between *C. moschata* and *A. thaliana*; The yellow lines indicated the syntenic *NHX* gene pairs between *C. maxima* and *A. thaliana*; The black lines indicated the syntenic *NHX* gene pairs between *C. pepo* and *A. thaliana*. All the data for the various species was extracted from *Cucurbit* genomics database.

MCScanX software was used to examine the collinearity and further investigate the evolutionary relationship between the *NHX* gene families in *C. moschata*, *C. maxima*, *C. pepo*, and *A. thaliana*. The findings revealed that, between *CmoNHXs* and *CmaNHXs*, *CpNHXs, AtNHXs*, 13, 11, and 5 syntenic gene pairs were identified, respectively (**Figure 4**; **Table S2**). In addition, between *CmaNHXs* and *CpNHXs*, *AtNHXs*, a total of 10, 4 syntenic gene pairs were discovered, respectively (**Figure 4**; **Table S2**). Two syntenic gene pairs between *CpNHXs* and *AtNHXs* were found (**Figure 4**, **Table S2**). We discovered that the homology of *C. moschata*, *C. maxima*, and *C. pepo* was substantially higher than that of *A. thaliana* based on the collinearity analysis of *NHX* genes in four species.

### Analysis of *Cis*-acting elements of *NHX* gene promoters in *Cucurbita*

**Figure 5:**
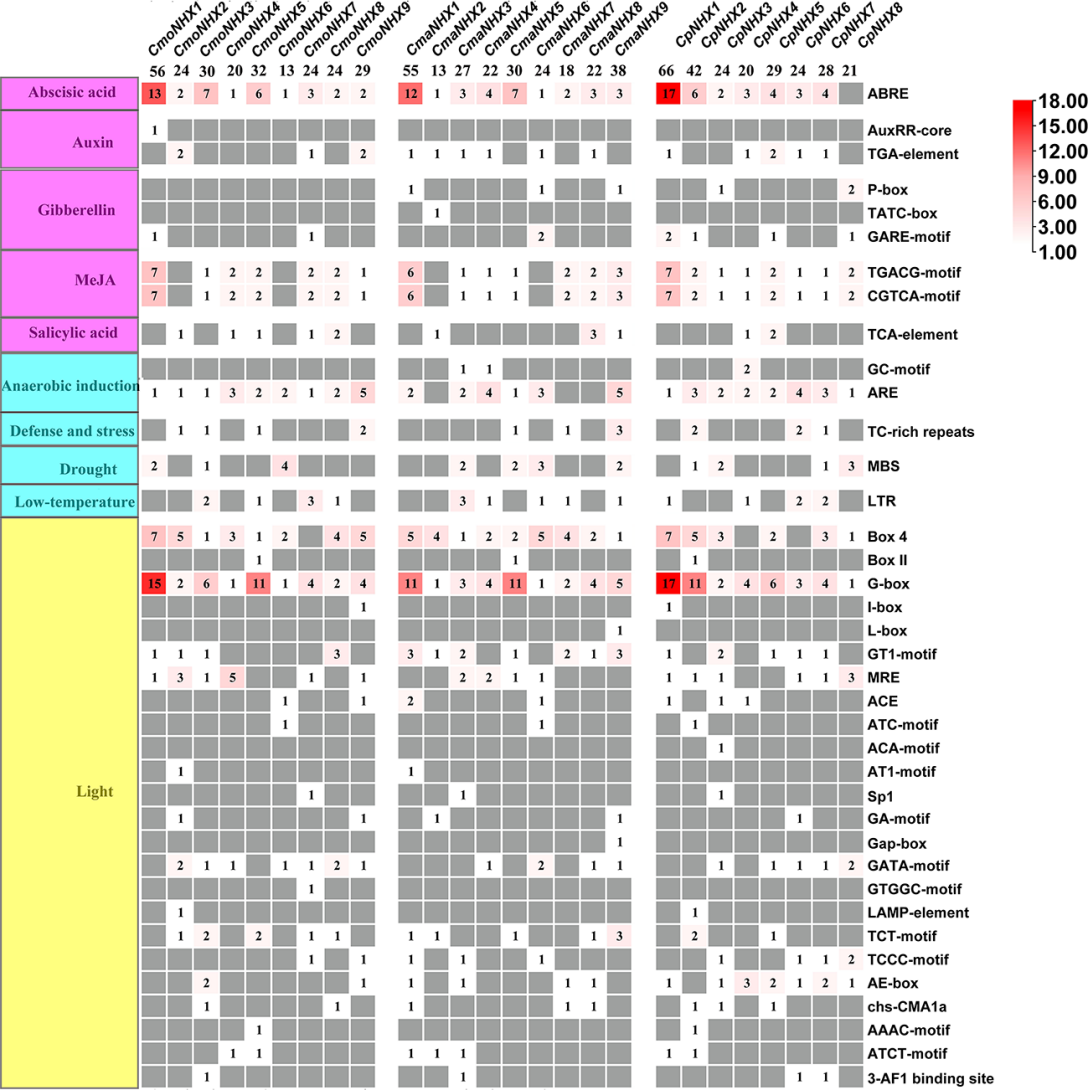
Analysis of promoter *cis*-acting elements of 26 *NHX* genes in *Cucurbita* genus. The leftmost side represents the functional distribution of *cis*-acting elements; the rightmost side represents the name of *cis*-acting element.

This study examined the *CmoNHX*, *CmaNHX*, and *CpNHX* promoter sequences and discovered 755 *cis*-acting elements in the promoter regions of 26 *NHX* genes. They included *CmoNHXs*, *CmaNHXs*, and *CpNHXs*, which each had 252 *cis*-acting elements, 249 *cis*-acting elements, and 254 *cis*-acting elements. The majority of these *cis*-acting elements were connected to hormone response, abiotic stress, and growth and development factors (**Figure 5**; **Table S3**). We focused on examining the *cis*-acting elements associated with salt stress because of the significance of the *NHX* gene under salt stress. Although ABRE, TGA-element, TGACG/CGTCA-motif are related to hormone response, G-box and GT1-motif are related to light response, MBS is related to drought stress response (**Figure 5**; **Table S3**), but related studies have found that these *c*is-acting elements are all involved in the response to salt stress (Yamniuk and Vogel, 2004; Saeediazar et al., 2014). In the present study, we discovered that the *CmoNHX1*, *CmaNHX1*, and *CpNHX1* promoters contain the most ABRE, G-box, and TGACG/CGTCA elements (**Figure 5**; **Table S3**). On the basis of the aforementioned analysis, we hypothesized that the genes *CmoNHX1*, *CmaNHX1*, *CpNHX1*, *CmoNHX5*, *CmaNHX5*, and *CpNHX5* may be crucial in salt stress.

### Response of *NHX* genes in *Cucurbita* to NaCl stress

**Figure 6:**
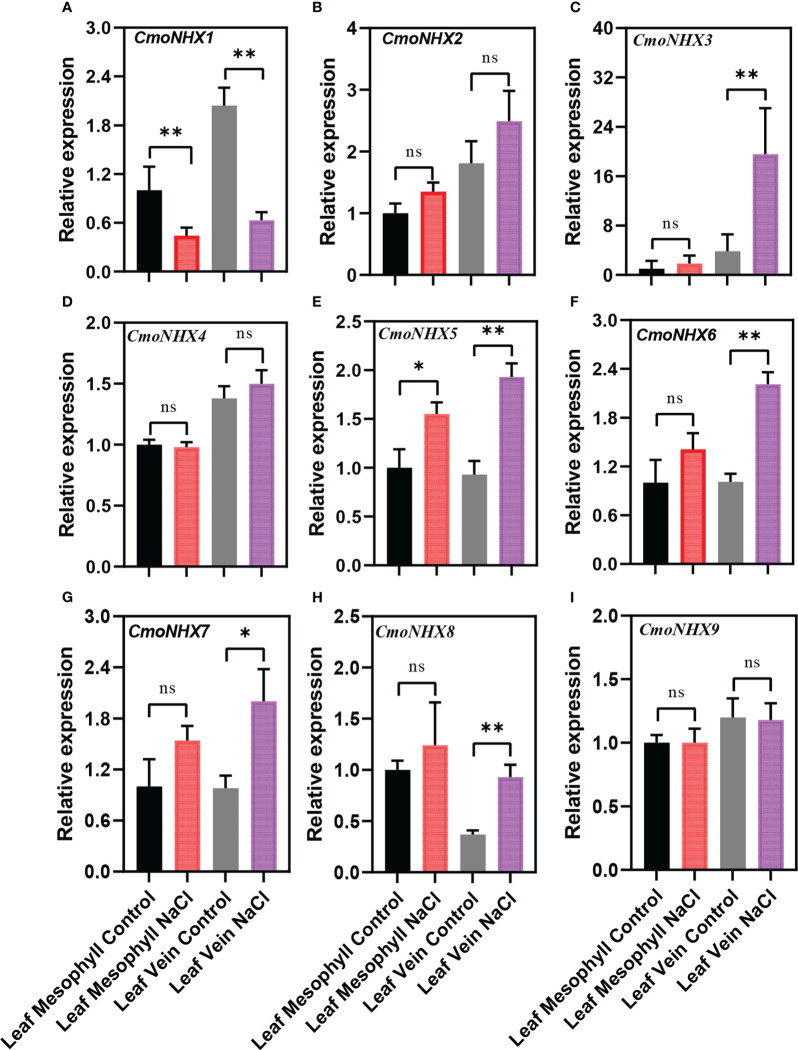
Relative expression of *NHX* genes from the leaf mesophyll and leaf veins of *C. moschata* (“*Rifu*”) in response to treatment with 100 mM NaCl for 24 h. Each data represents the average from three samples. The error bars represent the SDs. ^*^ indicates significance at *p* < 0.05, ^∗∗^ indicates significance at *p* < 0.01, NS indicates no significance.

**Figure 7:**
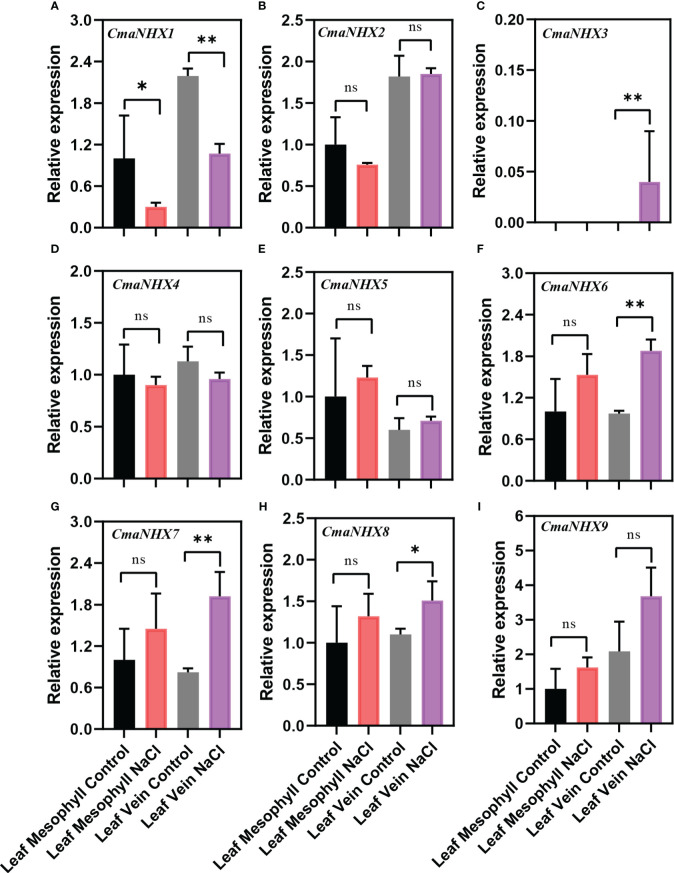
Relative expression of *NHX* genes from the leaf mesophyll and leaf veins of *C. maxima* (‘*Rimu*’) in response to treatment with 100 mM NaCl for 24 h. Each data represents the average from three samples. The error bars represent the SDs. ^∗^ indicates significance at *p* < 0.05, ^∗∗^ indicates significance at *p* < 0.01, NS indicates no significance.

The different expression patterns of *NHX* genes in *C. moschata* and *C. maxima* suggest that they might have different mechanisms to improve salt tolerance under salt stress. After NaCl treatment, the transcription levels of *CmoNHX2*, *CmoNHX5*, *CmoNHX6*, and *CmoNHX7* genes increased greatly in the mesophyll and vein of *C. moschata*, while the transcription level of *CmoNHX1* genes fell dramatically (**Figure 6**). For instance, after NaCl treatment, the transcription levels of *CmoNHX1* in the mesophyll and veins were considerably decreased by 55.44% and 69.04%, respectively, in comparison to the control treatment (**Figure 6**). In mesophyll, there was no apparent change between the NaCl treatment and the control treatment in the transcript levels of any *CmaNHX* genes. The transcription levels of *CmaNHX6*, *CmaNHX7*, and *CmaNHX8* under salt stress were significantly higher than those under control treatment, and the transcription level of *CmaNHX1* under salt stress was 51.26% of that under control treatment, despite the fact that *CmaNHX2*, *CmaNHX3*, *CmaNHX4*, *CmaNHX5*, and *CmaNHX9* did not change significantly under salt stress in the veins (**Figure 7**). These findings imply that *NHXs* might improve salt tolerance in two cultivars *via* various expression patterns. Overall, the transcriptional changes of *NHX* genes in response to salt stress provide insights into the molecular mechanisms of salt tolerance in *Cucurbita* cultivars.

### Response of *CmoNHX1*-heterologously expressed *Arabidopsis* plants to salt stress

**Figure 8:**
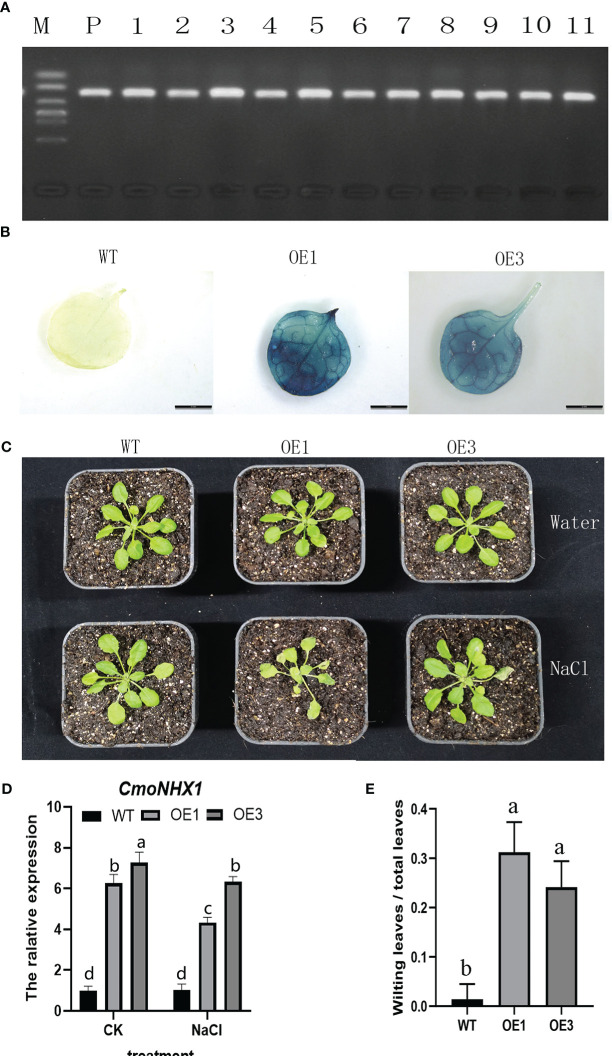
Positive identification and phenotype analysis of *CmoNHX1*-heterologously expressed *A. thaliana* plants. **(A)**, Identification of hygromycin resistance genes; **(B)**, GUS staining verification; **(C)**, Analysis of plant phenotype characteristics; **(D)**, Relative expression quantity of *CmoNHX1* in transgenic and wild-type plants; **(E)**, The ratio of wilting leaves to total leaves under salt stress. For the wild type, OE1 and OE3 plants, 8 plants were selected respectively for statistics. The data were represented as the means of three replicates, and error bars represented the standard deviations of means. Different letters above the bars indicated significant differences (*p* < 0.05) between plants.

Eight positive seedlings in the T1 generation were grown from *CmoNHX1*-heterologously expressed *A. thaliana* plants in order to investigate the function of *CmoNHX1*. On the leaves of T2 plants, we carried out hygromycin resistance gene identification (**Figure 8A**) and GUS staining validation (**Figure 8B**), after which we arbitrarily selected the OE-1 and OE-3 lines for additional investigation.

The transgenic *A. thaliana* plants and wild-type were both given NaCl treatments in order to study how the *CmoNHX1*-heterologously expressed *A. thaliana* plants responded to salt stress. Under water treatment, the phenotype revealed no discernible difference between transgenic plants and wild-type plants. However, when exposed to salt stress, the leaves of *A. thaliana* plants heterologously expressing *CmoNHX1* clearly yellowed and wilted in contrast to wild-type plants (**Figure 8C**). No significant difference between the transgenic lines OE1 and OE3 (**Figure 8E**). *CmoNHX1* expression under salt stress was considerably lower than it was after water treatment, according to qRT-PCR analyses (**Figure 8D**). These findings imply that *CmoNHX1* decreases the salt tolerance of plants under salt stress.

### Subcellular localization of CmoNHX1 protein

**Figure 9:**
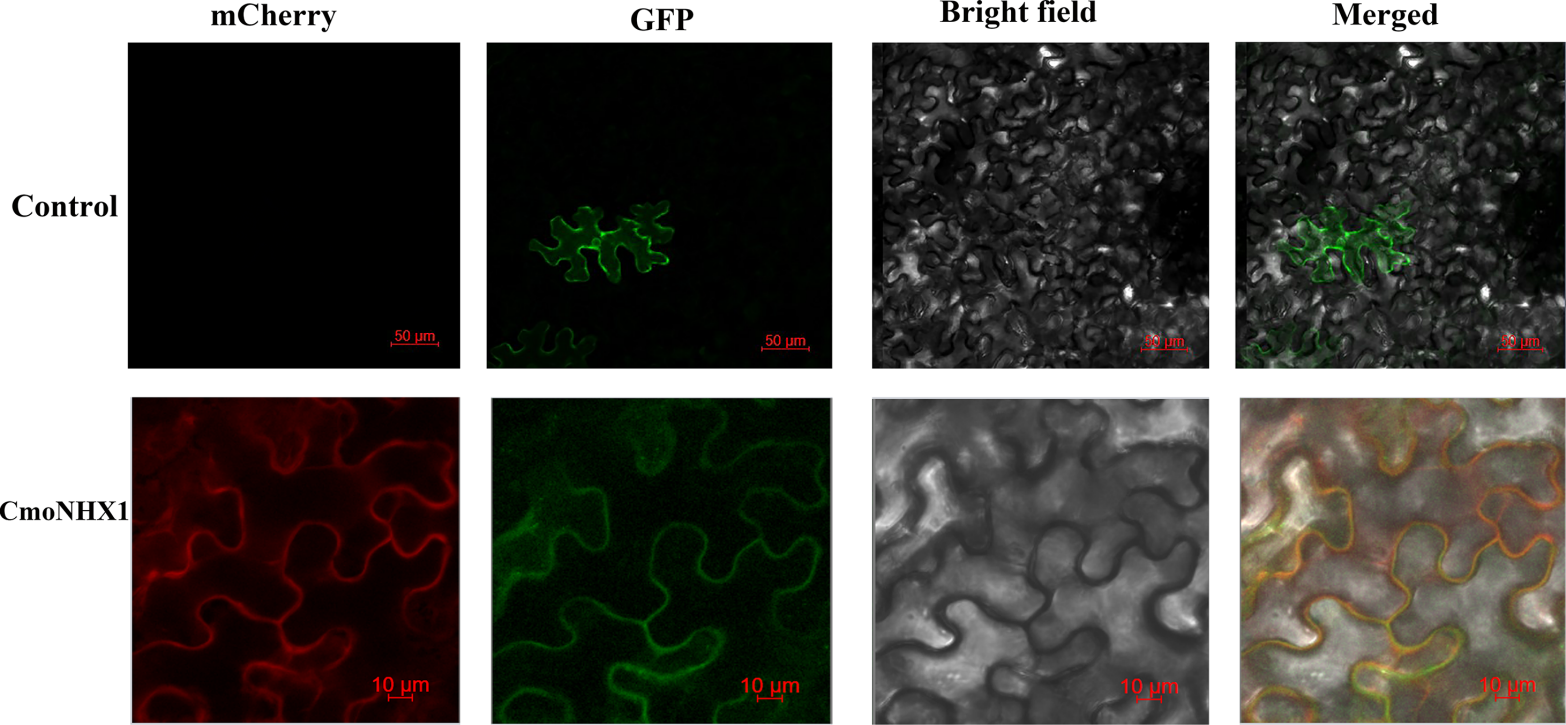
Subcellular localization of *CmoNHX1*. Transient expression of PRI101-GFP*-CmoNHX1* in tobacco leaf cells. The PRI101-GFP construct was used as the control. Control, PRI101-GFP; *CmoNHX1*, PRI101-GFP-*CmoNHX1*; mCherry, membrane marker: pm-rbCD3-1008; GFP, green fluorescence of *CmoNHX1*; Bright field, bright field images of tobacco leaf cells; Merged, overlay of bright field, green fluorescence and red fluorescence images; Bars 50/10 µm.

The discovery of the *CmoNHX1* gene’s temporary expression in tobacco demonstrated that GFP, a fusion protein created with CmoNHX1, emits light on the cytoplasmic membrane and overlaps with the red fluorescent protein of the membrane marker: pm-rbCD3-1008 (**Figure 9**). This suggests that CmoNHX1 is located on the cytoplasmic membrane.

## Discussion

The presence of *NHX* genes in various plant species indicates their important role in plant adaptation to stress conditions. The *NHX* gene family plays a vital role in salt tolerance and pH homeostasis by regulating ion transport in plants (Apse and Blumwald, 2007). Since the discovery of eight *NHX* genes in *A. thaliana*, researchers have found *NHX* gene families in other species, such as *P. trichocarpa* (Meng and Wu, 2018), *Zea mays* (Zhang et al., 2018), *B. vulgaris* (Wu et al., 2019), *Anemone vitifolia Buch* (Akram et al., 2020) and *Triticum aestivum* (Sharma et al., 2020). In this study, we discovered a total of 26 *NHX* genes from three cultivars of the *Cucurbita* genus. These genes’ physical characteristics, such as their number of amino acids, isoelectric point *pI*, *MW*, and transmembrane structure, were quite different from one another, indicating that these cultivars’ genetic diversity occurred during evolution (**Table 1**). The phylogenetic tree divided 26 NHX proteins into three subfamilies: Vac, Endo and PM. The Vac subfamily had the largest number of NHX proteins (**Figure 1**), which was similar to the results reported in *A. thaliana* (Philippe et al., 2012), *B. vulgaris* (Wu et al., 2019) and *P. trichocarpa* (Meng and Wu, 2018). This study’s findings showed that some *Cucurbita NHX* genes’ transcription levels changed under salt stress conditions, suggesting that these genes might be involved in the plants’ response to salt stress. The discovery of *NHX* genes in various plant species and their diverse physical characteristics highlights the importance of understanding the evolutionary history and genetic diversity of this gene family to elucidate its biological functions fully. Overall, this study provides valuable insights into the diversity and evolution of the *NHX* gene family in the *Cucurbita* genus, which could have important implications for developing salt-tolerant crops in the future.

In addition, motif analysis found that all NHX proteins contained Motif 11. Motif 7 and 12 only exist in the Vac subfamily. Motif 13 was also found only in PM and Endo subfamilies (**Figure 2C**). These results were consistent with those of PtNHX (Tian et al., 2017) and SbNHX (Kumari et al., 2018), indicating that the *NHX* family genes in *Cucurbita* were relatively conserved during evolution. Among the 26 *NHX* genes in *Cucurbita*, the number of exons from PM subfamily was 20 to 23, which was much larger than that in Vac subfamily, which was consistent with the report in *P. trichocarpa* (Tian et al., 2017) and *Glycine max* (Chen et al., 2015). This indicates that the genes of different subfamilies in the same species have structural diversity, so functional diversity may exist. The circular evolutionary tree divides CmoNHXs into three subgroups, with CmoNHX1 located in vacuole (Vac) subfamily but not the plasma membrane (PM) subfamily. However, the transient expression of CmoNHX1 protein in tobacco indicates that CmoNHX1 is located in the cell membrane. These two results are inconsistent, and we speculate that there may be temporally or spatially differentially expressed C*moNHX1*.

The primary driving factor behind plant genome evolution is the duplication of single genes, chromosomes, or entire genomes (Paterson et al., 2012). Since *NHX* genes were duplicated and lost during evolution, plants with the same ancestor have different numbers of *NHX* genes today. The genes of *C. moschata* and *C. maxima* essentially mapped to the same chromosome position (**Figure 4**), indicating that they may have shared an ancestor (Qanmber et al., 2019), and their evolutionary relationship is closer than that of *C. pepo*. Nine each and eight *NHX* genes were found in this study from three *Cucurbita* cultivars, respectively. Collinearity analysis is a tool that can be used to identify the evolutionary relationship between different species or cultivars based on shared gene sequences or synteny. In this case, the collinearity study suggests that *C. moschata* and *C. maxima* are more closely related to each other than they are to *C. pepo*, and that the number of shared syntenic genes between them decreases as the evolutionary distance between them increases. This information can help researchers to better understand the genetic basis of traits that are shared between different cultivars, as well as the genetic mechanisms that underlie the differences in those traits that have arisen during evolution. The findings of the collinearity study revealed that *C. moschata* and *C. maxima* shared 13 pairs of syntenic genes, whereas *C. moschata*, *C. maxima*, and *C. pepo* shared 11 and 10 pairs of syntenic genes, respectively (**Figure 5**). Before going through two independent phases of diversification, *C. pepo* originally underwent multiple domestication events in Mexico and the US. (Kates et al., 2017). According to available data, the *C. pepo* developed at around 30 ± 4 Mya (Schaefer et al., 2009). However, between 26 Mya and 3 Mya, C. moschata and C. maxima diverged from one another (Sun et al., 2017). This suggests that *C. pepo*, *C. moschata*, and *C. maxima* evolved at different rates and that the loss of gene segments or the translocation of chromosomes happened during the evolution process.

The main switch in gene transcription regulation, the *cis*-acting element, regulates a number of biological processes, including hormone response, abiotic stress response, and developmental process (Ding et al., 2018). Different *cis*-acting elements play specific functions in gene expression in plants. For instance, according to Ulmasov et al. (1997), auxin induction is primarily mediated by the AuxRE, DR5, AuxRR-core, and TGA-element motifs, while abscisic acid induction is frequently mediated by the ABRE motif (Shu et al., 2019; Wang and Huang, 2019). Inducing drought typically involves MBS and LTR (Dunn et al., 1998; Sazegari et al., 2015). In order to induce light, G-box, AT-rich, GT1-motif, and I-box elements are primarily used (Gilmartin et al., 1992). Furthermore, it was discovered in similar studies that the GT1-motif and TGACG-motif were identified as salt stress response elements (Büyük et al., 2016), and that the ABRE, G-box, MBS, and TGA-element had regulatory effects in salt stress situations (Saeediazar et al., 2014). The number of ABRE in *CmoNHX1* was 13. CmoNHX1 contains 15 G-boxes and has seven TGACG/CGTCA elements. Besides, to determine the response of *CmoNHXs* to salt stress, we excavated the transcriptome data (BioProject: PRJNA464060) published in 2018 (Niu et al., 2018) and analyzed the transcription profile of *NHXs* in the leaf mesophyll and leaf vein of the *C. moschata* cultivar, “*Rifu*” under salt stress. Results revealed that after NaCl treatment, the transcription levels of *CmoNHX1* in the mesophyll and veins were considerably decreased by 55.44% and 69.04%, respectively, in comparison to the control treatment (**Figure 6**). Based on the above analysis, *CmoNHX1* may be important players in salt stress, according to research on the NHX family’s potential involvement in a variety of biological processes. We looked at the phenotypes of wild-type and *CmoNHX1*-heterologously expressed *A. thaliana* plants in this study and discovered that there was no discernible difference between transgenic and wild-type plants under water treatment. However, when exposed to salt stress, the leaves of *CmoNHX1*-heterologously expressed *A. thaliana* plants clearly yellowed and wilted in contrast to wild-type plants (**Figure 8C**), indicating that *CmoNHX1* decreased plants’ ability to withstand salt. Additionally, qRT-PCR results demonstrated that *CmoNHX1* expression under salt stress was much lower than it was during water treatment (**Figure 8D**). Therefore, *CmoNHX1* mutants could be created using gene editing technologies in the future, improving the salt tolerance of *C. moschata* plants.

Under high salt treatment (200 and 300 mM NaCl), the transcription levels of *BvNHX3*, *BvNHX4*, and *BvNHX5* in barley leaves were substantially greater than those in roots. Additionally, researchers discovered that *BvNHX1* and *BvNHX3* isolated Na^+^ in vacuoles to lessen the harm that salt stress caused to plants (Wu et al., 2019). In this study, heterologous expression of *NHX1* in *A. thaliana* reduced plant tolerance to salt stress, a result consistent with the previous *cis*-acting element analysis and transcriptome data analysis, which demonstrated that *NHX1* negatively regulates salt tolerance in plants. The above result was contrary to the expression of *BnaNHX1* in cotton (Fu et al., 2020) and *BvNHX1* in barley (Wu et al., 2019). Based on the above results, we speculated that the mechanism of *NHX1* gene response to salt stress is different in different plant species.

## Conclusions

Overall, this study provides important insights into the *NHX* gene family in *Cucurbita* species and their response to salt stress. The identification of 26 *NHX* genes in three cultivars of the *Cucurbita* genus and the characterization of their physical and structural features, evolutionary relationships, and response to salt stress provide a comprehensive understanding of the *NHX* gene family in these species. The discovery of the importance of *CmoNHX1* in salt tolerance and the negative impact of its overexpression on salt tolerance also has practical implications for breeding salt-tolerant *Cucurbita* varieties. The findings of this study can serve as a foundation for further functional validation and exploration of the molecular mechanisms underlying the role of *NHX* genes in plant salt tolerance.

## Data availability statement

The original contributions presented in the study are included in the article/**Supplementary Material**. Further inquiries can be directed to the corresponding author.

## Author contributions

CS: Conceived and designed the research, basic bioinformatics analysis, revised the article. JY: Conceived and designed the research, basic bioinformatics analysis, wrote the original draft of the manuscript. XL: Vector construction, genetic transformation, positive identification of transgenic plants, qRT-PCR analysis of related Genes. RC: Vector construction, genetic transformation, positive identification of transgenic plants, qRT-PCR analysis of related genes. DL: Vector construction, genetic transformation, positive identification of transgenic plants. FW: Provides tools for bioinformatics analysis. XL: Provides methods for identification of transgenic plants. XZL: Revised the article. All authors contributed to the article and approved the submitted version.
