# Supplementary figures and images for: Genome-wide identification of NHX (Na+/H+ antiporter) gene family in Cucurbita L. and functional analysis of CmoNHX1 under salt stress
Source: Front Plant Sci. 2023 Mar 14;14:1136810. doi: 10.3389/fpls.2023.1136810 (PMC10043322; doi:10.3389/fpls.2023.1136810)

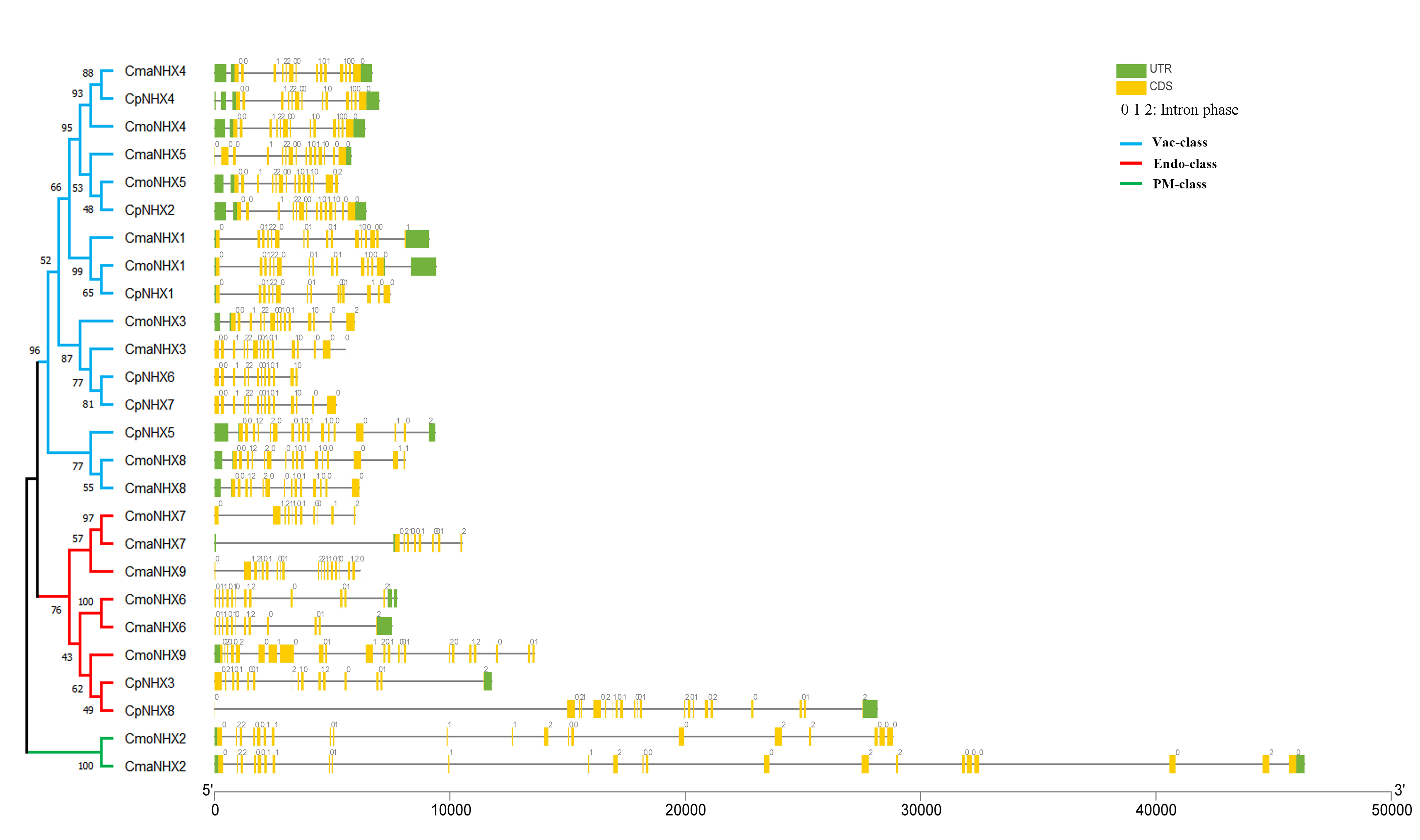

Supplement: Supplementary file 2 [file Image_1.tif]

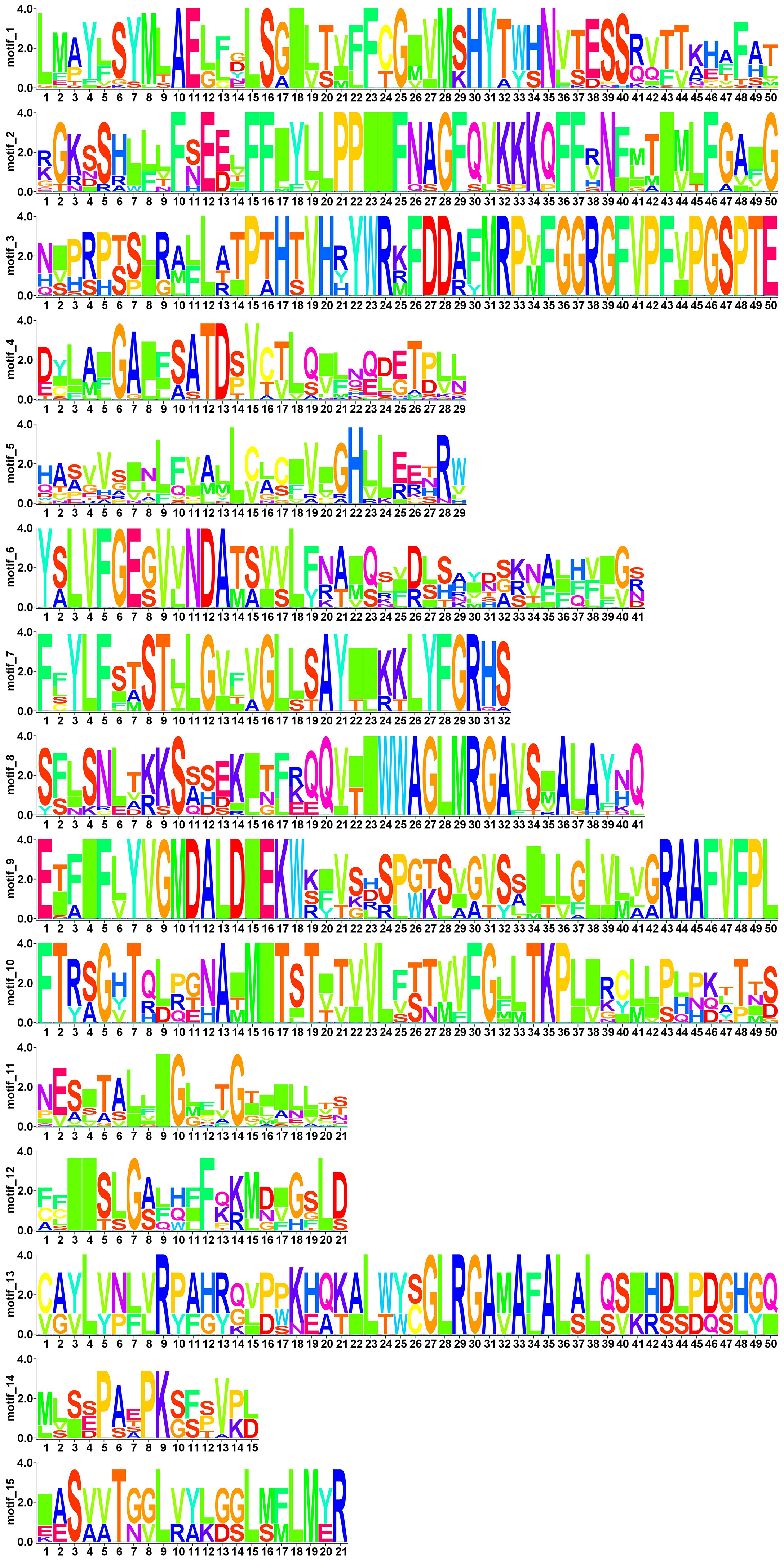

Supplement: Supplementary file 3 [file Image_2.tif]
